# Genome Sequence of SN1, a Bacteriophage That Infects Sphaerotilus natans and Pseudomonas aeruginosa

**DOI:** 10.1128/mra.00478-22

**Published:** 2022-08-03

**Authors:** K. M. Damitha Gunathilake, Denise M. Tremblay, Pier-Luc Plante, Ellen C. Jensen, Kenneth W. Nickerson, Sylvain Moineau

**Affiliations:** a Département de biochimie, de microbiologie et de bio-informatique, Faculté des sciences et de génie, Université Laval, Québec City, Québec, Canada; b Groupe de recherche en écologie buccale, Faculté de médecine dentaire, Université Laval, Québec City, Québec, Canada; c Institute of nutrition and functional foods, Université Laval, Québec City, Québec, Canada; d Félix d’Hérelle Reference Center for Bacterial Viruses, Université Laval, Québec City, Québec, Canada; e School of Biological Sciences, University of Nebraska, Lincoln, NE, USA; Portland State University

## Abstract

Phage SN1 infects Sphaerotilus natans and Pseudomonas aeruginosa strains. Its genome consists of 61,858 bp (64.3% GC) and 89 genes, including 32 with predicted functions. SN1 genome is very similar to Pseudomonas phage M6, which contains hypermodified thymidines. Genome analyses revealed similar base-modifying genes as those found in M6.

## ANNOUNCEMENT

Phage SN1 was isolated in 1979 from activated sludge samples obtained from a wastewater treatment plant (Lincoln, Nebraska, USA) using *S. natans* ATCC 13338 as the host (1, 2). An early study showed that the siphophage SN1 has unusual bases in its genome as confirmed by cellulose thin-layer chromatography (1). Its genomic DNA also showed resistance to type II restriction endonucleases (2). Host range studies indicate that phage SN1 can also infect Pseudomonas aeruginosa strains PAO33 and OT684 (2).

Here, phage SN1 was amplified with its host *S. natans* ATCC 13338 in nutrient broth (3 g/L beef extract, 5 g/L peptone) and agitated at 30°C (2). Cell debris were removed by filtration (0.45 μm) and filtrates were stored at 4°C until use. Phage SN1 also infected P. aeruginosa PAO1 (HER1153) in TSB/TSA medium at 30°C using both plaque assays and lysis of liquid cultures. Species identification of the above two host strains was confirmed by 16S sequencing.

Phage genomic DNA was purified from lysate (*S. natans* as host) using the phenol-chloroform extraction method (3). Library preparation for sequencing was carried out with Nextera XT DNA Sample Preparation kit (Illumina). A total of 186,025 paired-end reads (250 bp) were generated using the Illumina MiSeq Platform with Reagent kit v2. Read quality was evaluated with FastQC (4). Illumina adapters were removed and reads trimmed using Trimmomatic v0.39 (5). Trimmed reads assembly was performed by Spades assembler v3.13.0 (6). Two assemblies from independent lysates generated identical contigs of 61,858 nucleotides (218× and 170× coverage, respectively) with a GC content of 64.3%. Gene prediction and functional annotation were performed using RAST v2.0 (7) in combination with NCBI domain searches (https://www.ncbi.nlm.nih.gov/Structure/cdd/wrpsb.cgi) and blastp (https://blast.ncbi.nlm.nih.gov/Blast.cgi) (8, 9) using NCBI non redundant and nucleotide databases. Comparisons with other phage genomes were carried out with NCBI blastn (8). Bioinformatic tools were run with default parameters.

Annotation of phage SN1 genome predicted 89 genes and 32 predicted functions, which included proteins involved in nucleotide synthesis modification, genome replication, structural proteins, and cell lysis. The top hits for similar genomes consisted of several Pseudomonas phages with 95 to 98% nucleotide identity (73 – 96% query cover). Interestingly, phage SN1 has 96.76% nucleotide identity (91% query cover) with Pseudomonas phage M6 genome, which contains hypermodified thymines (reviewed in reference [10]). Half of the thymine residues in the M6 genome contain moieties synthesized through postreplicative modifications of 5-hydroxymethyl uridine. In M6-like phages, including SN1, the thymidine modification pathway includes several genes located upstream of the DNA polymerase gene (10). This cassette consists of genes that code for pyrimidine hyroxymethylase (Locus tag SN1_071), Nmad5 (SN1_019), aGPT-Pplase1 (SN1_020), nucleotide kinase (SN1_021), rSAM (SN1_022), pyridoxal-5′-phosphate (PLP) dependent enzyme (SN1_023), and aGPT-Pplase2 (SN1_024). The hypermodified thymidines likely explain the resistance of the SN1 genome to certain type II endonucleases (2). Finally, we observed a gene that codes for a putative antirestriction protein (Locus tag SN1_075). These proteins typically mimic the DNA structure and block type I restriction enzymes (11–12).

### Data Availability.

Genome sequence is available under GenBank number ON165687. Raw sequence reads are available under SRA number SRR18758685. Phage SN1 is available at www.phage.ulaval.ca.

## References

[1] Winston V, Thompson TL. 1979. Isolation and characterization of a bacteriophage specific for *Sphaerotilus natans* which contains an unusual base in its deoxyribonucleic acid. Appl Environ Microbiol 37:1025–1030. doi:10.1128/aem.37.5.1025-1030.1979. 485135 PMC243342

[2] Jensen EC, Schrader HS, Rieland B, Thompson TL, Lee KW, Nickerson KW, Kokjohn TA. 1998. Prevalence of broad-host-range lytic bacteriophages of *Sphaerotilus natans*, *Escherichia coli*, and *Pseudomonas aeruginosa*. Appl Environ Microbiol 64:575–580. doi:10.1128/AEM.64.2.575-580.1998. 9464396 PMC106085

[3] Moineau S, Pandian S, Klaenhammer TR. 1994. Evolution of a lytic bacteriophage via DNA acquisition from the *Lactococcus lactis* chromosome. Appl Environ Microbiol 60:1832–1841. doi:10.1128/aem.60.6.1832-1841.1994. 16349277 PMC201570

[4] Andrews S. 2010. FastQC: a quality control tool for high throughput sequence data. http://www.bioinformatics.babraham.ac.uk/projects/fastqc.

[5] Bolger AM, Lohse M, Usadel B. 2014. Trimmomatic: a flexible trimmer for Illumina sequence data. Bioinformatics 30:2114–2120. doi:10.1093/bioinformatics/btu170. 24695404 PMC4103590

[6] Bankevich A, Nurk S, Antipov D, Gurevich AA, Dvorkin M, Kulikov AS, Lesin VM, Nikolenko SI, Pham S, Prjibelski AD, Pyshkin AV, Sirotkin AV, Vyahhi N, Tesler G, Alekseyev MA, Pevzner PA. 2012. SPAdes: a new genome assembly algorithm and its applications to single-cell sequencing. J Comput Biol 19:455–477. doi:10.1089/cmb.2012.0021. 22506599 PMC3342519

[7] Aziz RK, Bartels D, Best AA. et al. 2008. The RAST Server: rapid annotations using subsystems technology. BMC Genomics 9:75. doi:10.1186/1471-2164-9-75. 18261238 PMC2265698

[8] Johnson M, Zaretskaya I, Raytselis Y, Merezhuk Y, McGinnis S, Madden TL. 2008. NCBI BLAST: a better web interface. Nucleic Acids Res 36:W5–W9. doi:10.1093/nar/gkn201. 18440982 PMC2447716

[9] Pruitt KD, Tatusova T, Maglott DR. 2005. NCBI Reference Sequence (RefSeq): a curated non-redundant sequence database of genomes, transcripts and proteins. Nucleic Acids Res 33:D501–D504. doi:10.1093/nar/gki025. 15608248 PMC539979

[10] Lee Y-J, Dai N, Müller SI, Guan C, Parker MJ, Fraser ME, Walsh SE, Sridar J, Mulholland A, Nayak K, Sun Z, Lin Y-C, Comb DG, Marks K, Gonzalez R, Dowling DP, Bandarian V, Saleh L, Corrêa IR, Weigele PR. 2021. Pathways of thymidine hypermodification. Nucleic Acids Res 50:3001–3017. doi:10.1093/nar/gkab781. PMC8989533 34522950

[11] Bandyopadhyay PK, Studier FW, Hamilton DL, Yuan R. 1985. Inhibition of the type I restriction-modification enzymes EcoB and EcoK by the gene 0.3 protein of bacteriophage T7. J Mol Biol 182:567–578. doi:10.1016/0022-2836(85)90242-6. 2989534

[12] Tock MR, Dryden DTF. 2005. The biology of restriction and anti-restriction. Curr Opin Microbiol 8:466–472. doi:10.1016/j.mib.2005.06.003. 15979932

